# Sacroiliac Joint Ankylosis in Young Spondyloarthritis Patients Receiving Biologic Therapy: Observation of Serial Magnetic Resonance Imaging Scans

**DOI:** 10.1002/art.40750

**Published:** 2019-02-14

**Authors:** Timothy J. P. Bray, Andre Lopes, Corinne Fisher, Coziana Ciurtin, Debajit Sen, Margaret A. Hall‐Craggs

**Affiliations:** ^1^ University College London London UK

## Abstract

**Objective:**

To assess the temporal relationship between initiating biologic therapy and magnetic resonance imaging (MRI) scores of inflammation and structural damage in young patients with spondyloarthritis.

**Methods:**

A local adolescent/young adult patient rheumatology database was searched for patients ages 12–24 years who had evidence of sacroiliitis on MRI and a clinical diagnosis of enthesitis‐related arthritis (ERA) with axial involvement or nonradiographic axial spondyloarthritis. Patients treated with tumor necrosis factor inhibitor (TNFi) therapy who had undergone a minimum of 1 MRI scan before and 2 MRI scans after starting TNFi therapy (over ≥2 years) were included. Images of the sacroiliac joints were scored for inflammation and structural abnormalities (including erosions, fat metaplasia, and fusion). The effects of TNFi therapy and of time since initiation of TNFi therapy on inflammation and structural abnormalities were assessed using a mixed‐effects regression analysis.

**Results:**

Twenty‐nine patients (ages 12–23 years) with ERA or nonradiographic axial spondyloarthritis who underwent TNFi therapy were included. Inflammation scores were significantly lower in patients receiving TNFi treatment (*P* = 0.013), but there was no significant effect of time from TNFi initiation on inflammation (*P* = 0.125). Conversely, there was no significant effect of active TNFi treatment on fusion scores (*P* = 0.308), but fusion scores significantly increased with time from TNFi initiation (*P* < 0.001); a similar positive relationship between time since biologic start and fat metaplasia scores was observed.

**Conclusion:**

TNFi therapy failed to prevent the eventual development of joint ankylosis in this cohort of young patients with spondyloarthritis, despite a substantial reduction in inflammation with TNFi therapy.

## Introduction

The spondyloarthritides are a group of immune‐mediated inflammatory disorders that are characterized by inflammation of the spine, entheses, and peripheral joints. Ankylosing spondylitis (AS) is the “prototypic” spondyloarthritis and causes ankylosis (fusion) of the axial skeleton with subsequent disability, morbidity, and impaired quality of life 1. However, ankylosis may be less severe or absent in other spondyloarthritis subgroups, suggesting that these groups represent a milder or earlier form of the disease 1. For example, patients with nonradiographic axial spondyloarthritis have little or no structural damage, and pediatric patients with spondyloarthritis—who are typically diagnosed as having enthesitis‐related arthritis (ERA)—may have a similar phenotype 2, 3. However, it remains unclear whether the disease in these patients represents an early form of AS or a fundamentally different subtype of spondyloarthritis. Furthermore, it is not known whether treating these patients early in their disease might prevent ankylosis.

Evidence from the preclinical literature suggests that new bone formation may be initiated by an initial inflammatory trigger 4, 5, and data from cohorts of adult patients with spondyloarthritis suggest that structural damage is often preceded by inflammation 6, 7. These findings suggest that early, aggressive treatment of spondyloarthritis might reduce the stimulus for subsequent ankylosis 8. However, studies of animal models of the spondyloarthritides have unravelled alternative pathways, suggesting that an uncoupling of inflammation and bone formation, each involving different molecular mechanisms, may take place, with bone morphogenetic protein and Wnt signaling involved in the bone formation pathway 9. In adults with established AS, clinical trials of tumor necrosis factor inhibitor (TNFi) therapy have produced mixed results, but have not shown a clear reduction in spinal radiographic progression when compared to historical cohorts of patients who have never been treated with a TNFi 10, 11.

Despite their well‐recognized efficacy in controlling inflammation, the debate about the role of TNFi as disease modifiers in spondyloarthritis continues. In this study, we assessed serial magnetic resonance imaging (MRI) scans in young patients with axial spondyloarthritis undergoing biologic therapy, in order to describe the changes in inflammation and structural damage occurring over time.

## Materials and methods

This retrospective study was covered by Institutional Review Board approval from the National Research Ethics Service Committee London (Bentham, UK) (reference no. 11/LO/0330). Informed consent was waived because of the retrospective nature of the study.

A local rheumatology database containing clinical data from adolescents and young adults was used to identify patients ages 12–24 years who had evidence of sacroiliitis on MRI, and who had a clinical diagnosis of ERA with axial involvement or nonradiographic axial spondyloarthritis 2, 3. A subsequent search of the picture‐archiving and communication system was used to identify all patients who had undergone at least 3 MRI scans of the sacroiliac joints over at least a 2‐year period, with at least 1 scan conducted before or at the time of starting the TNFi (adalimumab, etanercept, or infliximab) therapy, and at least 2 scans conducted after the start of the TNFi therapy. Posttreatment scans were performed either due to clinical need or as a part of routine imaging in the follow‐up period, according to a previously described protocol 12.

**Figure 1:**
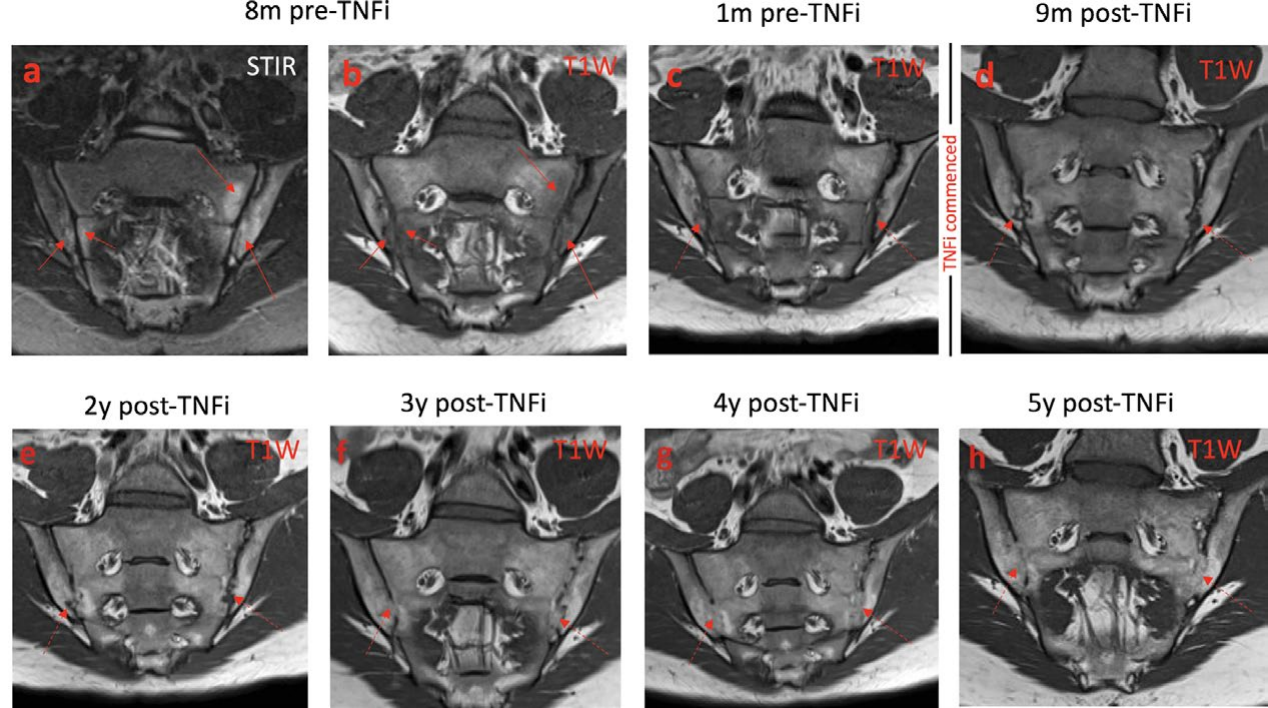
Magnetic resonance images from a representative patient showing the progression of ankylosis over a 5‐year period. a–c, Before the initiation of tumor necrosis factor inhibitor (TNFi) therapy, bilateral bone marrow edema is evident on the STIR image (a) and T1‐weighted (T1W) images (b and c). d–h, After TNFi therapy, the joint erosions gradually become less distinct, and the joints ultimately fuse in anatomic locations similar to those in which the initial edema was found. Arrows indicate the areas of edema (on STIR images) and fat metaplasia (on T1W images).

The posttreatment protocol was as follows: MRI scans were acquired on a 1.5‐Tesla MRI system with integrated posterior and anterior surface coils, using a specified protocol that included STIR, T1‐weighted turbo spin‐echo (T1WSE), and post–gadolinium‐enhanced fat‐saturated images of the sacroiliac joints and thoracolumbar spine. Sequence parameters included the following: for STIR images, repetition time (TR) 4,340–6,070 msec, echo time (TE) 83 msec, and inversion time (TI) 150 msec; for T1WSE images, TR 475–610 msec and TE 11 msec; and for T1WSE images with fat saturation, TR 619–715 msec and TE 11 msec. Sacroiliac joint images were acquired in both para‐coronal (angled parallel to the sacrum) and para‐axial (angled perpendicular to the sacrum) planes, and thoracolumbar spine images were acquired in the sagittal plane with extended lateral coverage. Only the sacroiliac joint images were used for the current analyses. Representative MR images demonstrating the progression of ankylosis in a young patient over a 5‐year period, before and after TNFi therapy, are shown in Figure 1.

MR images of the sacroiliac joints were scored independently by 2 readers (TJPB and MHC) with specific expertise in musculoskeletal imaging and in the assessment of spondyloarthritis. The extent of inflammation was assessed on STIR images using the Spondyloarthritis Research Consortium of Canada scoring system 13, taking care to differentiate between areas of high signal due to skeletal immaturity and bone marrow edema. Chronic inflammatory (structural) abnormalities were assessed on T1‐weighted images using a recently proposed structural score, which measures the extent of erosions, fat metaplasia, and joint fusion 14. For each subject, the images were scored in temporal order to enable comparison with previous scans. Readers were blinded with regard to the biologic start dates and to the clinical status at the time of each scan.

The statistical analysis plan was designed to evaluate the effect of the biologic treatment as well as the effect of time from biologic initiation on 4 outcomes, which were measured using MRI scores. The outcomes assessed were MRI scores of inflammation, fat metaplasia, erosions, and fusion. For each outcome, we took the mean of the 2 observers’ measurements for the statistical analysis. A mixed‐effects model was used for each outcome and included, as explanatory variables, treatment (receving biologic therapy versus not receiving biologic therapy) and time from biologic start, and accounted for repeated measurements in each patient. Assuming a quadratic, cubic, and categorical relationship between time and the outcome measures based on likelihood ratio testing, the linear model was compared to more complex models. If this analysis did not reveal a significant improvement with any of the more complex models over the linear model at a 5% significance level, then the reference results were derived from the linear model. For completeness, the fluctuations over time in each of the MRI scores were summarized from the results derived from the saturated model, where time was included as a categorical variable.

We also tested for relationships between inflammation and fusion, between fat metaplasia and fusion, between erosions and fusion, and between inflammation and fat metaplasia, using random intercept and slope models for each of the relationships in question. For example, in order to assess whether, adjusting for time, inflammation scores have an effect on fusion, a random intercept and slope model was implemented in which we included fusion as the outcome variable and time and inflammation scores as the explanatory variables.

## Results

Twenty‐nine patients (23 male and 6 female) were included in the study. The mean age at biologic start was 17 years 2 months (range 12 years 3 months to 22 years 10 months). Twenty‐three patients were HLA–B27 positive, 2 were negative for HLA–B27, and 4 had not been tested. The mean symptom duration in patients at the time of biologic therapy start was 5 years 3 months (range 4 months to 11 years). In total, 18 patients were treated with etanercept, 8 with adalimumab, and 3 with infliximab. Five patients switched biologics during the follow‐up period of the study (due to side effects in 3 cases, and due to inefficacy in 2 cases). The mean number of MRI scans performed per patient was 4.5 (range 3–7), and the mean interval between the first and last scans was 5 years 2 months (range 1 year 9 months to 9 years 11 months).

Our analysis of the mixed‐effects model did not reveal a significant improvement with any of the more complex models over the linear model. Therefore, the linear model was selected for the main analysis.

**Figure 2:**
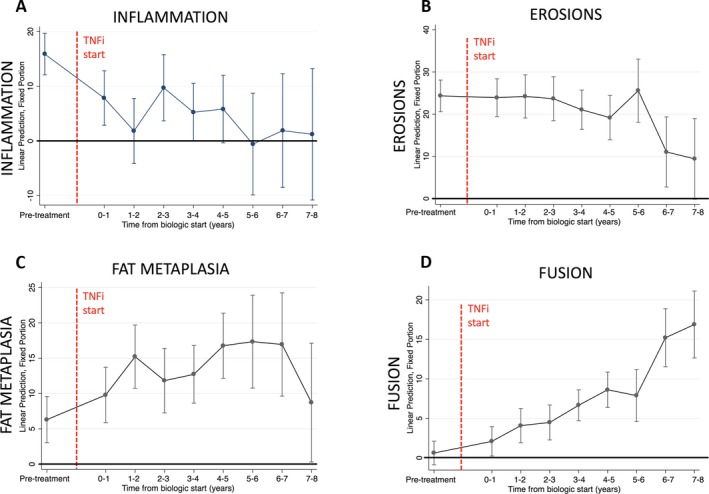
Scores of inflammation (A), erosions (B), fat metaplasia (C), and fusion (D) over time before and after the start of tumor necrosis factor inhibitor (TNFi) therapy, in the linear mixed‐effects model. Bars show the estimate for each time point ± 95% confidence interval.

Estimates of inflammation, erosions, fat metaplasia, and fusion scores in the 29 patients over time, derived from the saturated model for the purposes of illustration, are shown in Figures 2A–D. Inflammation scores were significantly lower in patients receiving TNFi treatment than in those not receiving TNFi treatment (β = −6.94, 95% confidence interval [95% CI] −12.4 to −1.44; *P* = 0.013), but there was no significant effect of time since biologic initiation on inflammation scores (β = −0.90, 95% CI −2.04 to 0.35; *P* = 0.125) (Figure 2A).

Conversely, there was no significant effect of active TNFi treatment on fusion scores (β = −0.74, 95% CI −2.17 to 0.69; *P* = 0.308), but there was a significant positive relationship between time since biologic start and fusion scores (β = 1.55, 95% CI 1.02 to 2.09; *P* < 0.001).

Similarly, there was no significant effect of active TNFi treatment on fat metaplasia scores (β = 1.66, 95% CI −1.38 to 4.70; *P* = 0.285), but time since biologic start was significantly associated with fat metaplasia scores (β = 1.53, 95% CI 0.57 to 2.50; *P* = 0.002) (Figure 2C). There was no significant effect of TNFi treatment (β = 0.12 −3.83 to 4.06; *P* = 0.954) or time since biologic initiation (β = −0.82, 95% CI −1.91 to 0.27; *P* = 0.139) on erosion scores (Figure 2B).

When the effects of time and treatment were accounted for, there was a significant negative relationship between inflammation and fat metaplasia scores (β = −0.25, 95% CI −0.34 to −0.17; *P* < 0.001). However, there was no significant relationship between inflammation and fusion scores (β = −0.02, 95% CI −0.06 to 0.03); *P* = 0.443), fat metaplasia and fusion scores (β = 0.04, 95% CI −0.04 to 0.12; *P* = 0.305), or erosions and fusion scores (β = −0.05, 95% CI −0.11 to 0.01; *P* = 0.104).

## Discussion

The spondyloarthritides encompass a wide range of disease phenotypes, each of which varies in terms of disease severity, the presence of structural damage, and age at presentation. Spondyloarthritis patients who present early might offer a unique opportunity to study the disease course, and to characterize the evolution of inflammatory and structural damage over time. In addition, it has been suggested that early intervention might help to avoid structural complications and thereby improve long‐term outcomes 8. However, we observed that TNFi therapy did not prevent the eventual fusion of the sacroiliac joints in patients with ERA, despite there being relatively little structural damage (particularly fusion) at presentation and despite the fact that inflammation was substantially reduced by TNFi treatment. Although sacroiliac joint ankylosis in itself may have a relatively small effect on disability, it is possible that the effect of treatment on inflammation and structural damage would be similar in the remainder of the spine.

The results of our study do not enable us to comment on the specific cause of fusion in these patients; we do not know whether fusion is a consequence of inflammation itself, or whether it is related to the TNFi therapy. Although the levels of inflammation were substantially reduced after treatment, it is possible that low levels of inflammation persist and drive joint ankylosis. Alternatively, joint fusion may be triggered by the initial inflammatory insult and then proceed independently, as other authors have suggested 4, 5. Either way, these results suggest that more research is needed into strategies for preventing structural damage, either through a reduction in inflammation or the inhibition of bone‐forming pathways.

Some limitations have arisen due to the retrospective nature of the study. Since the number of MRI scans was an inclusion criterion, it is likely that our cohort includes patients with more severe disease. However, the severity of the disease in this cohort of patients with “definite” spondyloarthritis probably also increased the size of the effects in question, which may have actually strengthened the analysis.

An additional issue is that scans were acquired at irregular intervals (as determined by clinical need), meaning that the data were not evenly distributed in the posttreatment period. Nonetheless, our regression model accounted for the clustered nature of the data, reducing any potential effect on the final analysis. It should be emphasized that spondyloarthritis in young people is comparatively rare, and it would have been very difficult to acquire data over such a long time period in a prospective manner.

The development of imaging methods that could identify new bone formation before the development of overt ankylosis might reduce the follow‐up period needed to assess the effect of novel therapies on new bone formation. Several groups have begun to explore MRI methods that can derive signal directly from mineralized bone or quantify bone mineral density indirectly 15, and it is likely that these techniques will become more widely applicable in the years to come.

In conclusion, we found that TNFi therapy in young patients with spondyloarthritis failed to prevent the eventual development of joint ankylosis, despite a substantial reduction in inflammation with TNFi therapy.

## Author Contributions

All authors were involved in drafting the article or revising it critically for important intellectual content, and all authors approved the final version to be published. Drs. Bray and Hall‐Craggs had full access to all of the data in the study and take responsibility for the integrity of the data and the accuracy of the data analysis.

### Study conception and design

Bray, Hall‐Craggs.

### Acquisition of data

Bray, Fisher, Ciurtin, Sen, Hall‐Craggs.

### Analysis and interpretation of data

Bray, Lopes, Hall‐Craggs.
